# Book Reviews

**Published:** 1811-05

**Authors:** 


### CRITICAL ANALYSIS

OF

### RECENT PUBLICATIONS

IN THE

DIFFERENT BRANCHES OF PHYSIC, SURGERY, AND MEDICAL PHILOSOPHY,

---

*Observations on the Natural History, Climate, and Diseases of Madeira, during a period of eighteen years. By* William Gourlay, M.D. Fellow of the Royal College of Physicians, Edinburgh; and Physician to the Factory at Madeira. 8vo. pp. 158. Callow, 1811.

From the opportunities for observation and practice which the author's long residence in Madeira has procured him, we anticipated copious and valuable information respecting an island of which we hear much and know little. When chagrined with want of success, and wearied with the progressive increase of all the patients' symptoms, we propose a voyage to Madeira as a last hope, although we are no further acquainted with the country, than having some confused notion that the climate is mild.

The first part of this volume treats of the discovery, situation, aspect, and soil of the island; of its vegetable and animal productions; and of the constitution, customs, and manners of the inhabitants.

"Madeira is situated in 32 degrees, 37 minutes, 30 seconds, north latitude, and in 17 deg. 5 min. longitude, west of Greenwich, about 80 leagues N. by E. from Teneriffe, 120 leagues from Cape Cantin, on the coast of Africa, nearly 100 leagues from the Isle of Terno, and about 17 leagues S. W. from Porto Santo. It is about 120 miles in circumference, its greatest length from E. to W. being 45 miles, its greatest breadth from S. to N. 15 miles, and its least breadth 8½ miles. It is formed of lofty mountains, of hills, and fruitful vallies, and in figure makes an oblong irregular quadrangle. Its capital is Funchal, which is situated on the south side of the island at the bottom of a spacious valley, open to the sea, and surrounded by lofty mountains, having all the appearance of an amphitheatre, gradually ascending to a great height. Its mountains and hills generally rise with a slow ascent, the highest points of land being about 8250 feet, or one mile and a half, above the level of the sea. The situation of Madeira, in some places, presents a most picturesque and enchanting appearance, while in others, huge perpendicular rocks, lofty precipices, prominent ridges, deep excavations and chasms, innumerable cascades, liberally supplied with rivulets, beautiful vallies, deep gullies and ravines, containing immense torrents of water, afford a highly varied, sublime, and no less alarming picture of nature." P. 5—6.

This island was formerly extremely fertile, and though the soil has been exhausted by a succession of crops for nearly four hundred years, without much assistance from culture, it still nourishes a variety of vegetable productions; all sorts of tropical, as well as European fruits, will flourish on this favoured spot, and every kind of grape grows there.

Cattle, poultry, fish, &c. &c. are very plentiful; and various species of small birds are seen. We wish that Dr. Gourlay had enabled us to detail more particulars of the natural history of Madeira; his information on this head is very scanty, although many rich and curious productions of nature are to be met with in the island.

His account of the inhabitants is interesting.

"The natives of Madeira, particularly the peasantry, (he observes) are distinguished by an olive or tawny colour of skin, and a swarthy complexion; nor is it improbable that they are of a Mulatto or Moorish origin. Indeed only a few of the first families at all resemble in complexion the fair inhabitants of northern Europe, and these are unequivocally of Portuguese extraction. The people of Madeira are, in general, athletic, well made, active, and of a middle stature. Those of the lower class, or the country people, are sober, inoffensive, economical, and capable of induring much hard labour; in the prosecution of which they are often reduced to great emaciation of body, and debility of constitution, and thus a premature old age is brought on. The higher classes, on the contrary, are inclined to corpulence, and at the same time more disposed to indolence, attended with a moroseness of temper, and disposition to melancholy: though sober in respect to drinking, they are apt too often to indulge in eating to excess; from this circumstance, joined to the sedentary life they lead, they become subject to a variety of chronic disorders, and also early arrive at a premature old age; nor is it to be concealed that of late years the use of spirituous liquors has become common here among all ranks, which has opened a new field for the production of a long train of maladies." P. 27—28.

The women marry early, are very prolific, and soon lose the charms of youth. Their habits are adverse to health, leading very sedentary lives, seldom going out but to church, and performing many religious exercises. The younger branches of the first families are immured within convents, from which, after taking the veil, they never issue.

The climate of Madeira forms the subject of the second part of this work, and extracts from a Meteorological Register, kept during a period of sixteen years, are inserted. The temperature appears to be pretty uniform, the hottest months being rendered pleasant by a succession of land and sea breezes. The following table from Kirwan's "Temperature of different latitudes," presents the average temperature of every month in the year.

"Madeira, Funchal, lat 32° 37', long. 17°, mean height of the thermometer for every month, taken from an average of four years observations.

| | | | |
|---|---|---|---|
| Jan. | 64,18. | July, | 73,45. |
| Feb. | 64, 3. | August, | 75,02. |
| March, | 65, 8. | Sept. | 75,76. |
| April, | 65, 5. | October, | 72, 5. |
| May, | 66,53. | Nov. | 69,08. |
| June, | 69,74. | Dec. | 65. |

The following is the average temperature of Madeira, compared with that of London, for the whole year, as well as during the coldest and warmest months, which are January and July. Taking the average temperature of London at 1000, the heat of Madeira is 1319. In January 0559, July 1128."

The third part treats of the diseases of Madeira; these the author arranges under the heads endemic and epidemic.

The first division comprehends various affections of the skin, complaints of the chest, rheumatism, colic, tetanus, dropsy, &c. Dr. Gourlay begins with describing elephantiasis, or Arabian leprosy, which is very common among the lower classes of people in Madeira. The disease is hereditary, and may be communicated from one person to another, although it does not appear to be of a very contagious nature.

"It generally shews itself by tubercles upon the face and upper extremities, and sometimes upon the trunk of the body and penis. Ill-conditioned ulcers of the legs also take place, in some instances, attended with acute pain; large indolent glandular tumours occupy the upper and anterior part of the thigh. The fingers become contracted, and the feet hard and swelled. The fingers also, and toes, are occasionally destroyed by ulceration; the same disposition to irregular tumours and ulceration attacks the throat.

"In those affected by the disease, previous to the age of puberty, the usual signs that mark this period of life do not appear. The beard, the usual sign of virility, is wanting; the hair is deficient on the pubis and scrotum, as well as on the axilla and breast. No desire prevails for the venereal passion: the voice preserves its puerile tone, and does not acquire the real strength and masculine expression. Even the testicles, not called upon for the exercise of their functions, gradually waste." P. 68—9.

Young females affected with this disease experience no increase or fulness of the breasts, no growth of the external parts of generation, no appearance of the menses, have no hair on the pubis or axilla, and feel no disposition to venery.

"Even where the disease makes its first attack, at a much later period, the marks of sexual maturity, which are already established, gradually disappear, and are attended with impotence or very impaired powers of generation.

These circumstances denote that the elephantiasis of Madeira differs from the same complaint in other countries: for almost every writer on the subject has described the unhappy victims of this disease, raging with insatiable irresistible desire for venery; *rabiosa inest libido.* In support of the contagious nature of this disease, Dr. Gourlay states, that

"About thirty years ago, in the village Ponta de Sol, fourteen miles distant from Funchal, the capital of the island, (Madeira) it raged with all the violence of an infectious malady, passing from one family to another, and threatening to extend its ravages into the neighbouring country, till the governor took this (the) prudent and wise step of separating the healthy from the diseased, and preventing their interference with each other." 72.

Some cases also under the author's immediate notice are cited in favour of this opinion. Where it affects people who have previously been healthy, the mode of attack resembles that which distinguishes contagious diseases. "It is ushered in with rigors and other symptoms of pyrexia, while at the same time there is no evidence of the presence of any other existing morbid cause." We have hitherto regarded this disorder as an incurable one, Dr. Gourlay however has occasionally succeeded in affording relief. In the incipient state, he has, in a few instances suspended the progress of the disease by the use of calomel. In other cases he found great benefit follow the internal administration of the lacerta (agilis) or common lizard.

"As a medicine, this reptile acts as a powerful stimulant on the living solid, opening the several excretions, and producing large evacuations, particularly by the skin and urine, which are at the same time not attended with any debilitating effect. By this mode of operation, it will be found to have the certain influence of arresting the progress of the worst symptoms of elephantiasis, if not the whole, and in many cases to have surprisingly restored parts, which for years had been morbidly enlarged, to their natural size, and even sensibility; though for that period they had continued in a torpid state. Its operation also seems to vary somewhat in different cases; at times the different secretions seem increased by it all at once, viz. the perspiration, urine, and saliva; at other times, merely an increase of saliva takes place." P. 74.

Diarrhœa sometimes followed the use of this remedy, and it occasionally produced vertigo. Some successful cases are subjoined from which we are convinced that this reptile possesses considerable efficacy in the cure of elephantiasis: It has also been administered with advantage, in herpes, chronic rheumatism, dropsy, and scrofula.

As most of the diseases in Madeira are similar to those of this country, we shall confine our extracts to such as present unusual appearances, appearances, or have some peculiar claim on our attention. Dr. Gourlay mentions an inveterate species of psora, termed by the natives, ouçao. It is well described by Dr. Adams in his work on morbid poisons. This affection usually attacks children at an early age, and is evidently occasioned by small animalcules piercing the skin. They possess the power of leaping like fleas; are somewhat larger than cheese mites, and belong to the genus acarus (siro exulcerans). Linn.

"The first appearance of the disease is in the form of a pellucid watery vesicle, attended with intolerable itching, and which, on being rubbed, breaks and discharges a thin watery fluid. A crust or scab is afterwards formed, from under which there is again emitted an acid ichorous matter. This matter corrodes the neighbouring parts, and tends to extend the disease, which is further assisted by the ova of the original infectious animalcules, and the locomotive power they are ascertained to possess."

The cure is most frequently performed by means of extraction with a pin or needle; but the complaint is easily removed by the application of mercurial and sulphur ointments.

"From a similar cause of animalcular irritation is derived another cutaneous malady, known by the name of alfora, from the small winged insect which occasions it. This insect is about the size of the one described, as producing the itch, and the affection it entails is most troublesome during harvest, and immediately after it." P. 85.

From cutaneous affections, our author proceeds to diseases of the chest, which in spite of its boasted salubrity are common in Madeira; the natives even are often affected with pulmonary consumption.

"Persons of all ages, and of both sexes, fall victims to it; nay, whole families have at times been suddenly swept away by it.

"The species of the disease that produces the ravages here, is that connected with scrofula, a disorder equally common here as in the colder regions of Europe: it uniformly at first assumes the appearance of a mild catarrh, but afterwards, when the real pulmonary symptoms commence, they prove more violent and rapid in their progress, than in the phthisis of northern climates."

An idea prevails in Madeira that this disease is of a contagious nature, and on that account the inhabitants of Funchal will hardly under any circumstances receive a phthisical patient into their houses.

The proper period for consumptive persons to leave England is the month of October, the fittest season in Madeira being from November to the beginning of June. In the treatment of phthisis, Dr. Gourlay experienced the most success from a cautious administration of digitalis. In the incipient stages, he generally effected a perfect cure; in the more advanced stages great great relief, and in some instances even complete restoration to health, by the use of this remedy.

In the second division, epidemics, the author classes those diseases which arise from a specific contagion. Fever is frequent in the island, and it usually assumes the typhoid character. Dr. G. attributes the fatality of this complaint to bad practice, and especially to bleeding. This is often the case in the country; but in towns, under regular practitioners, the termination is generally favourable.

In 1806, scarlatina was epidemic the first time in Madeira, and attended with a great fatality.

"The characteristic symptoms which marked its attack, were inflammation of the tonsils, and mucous membrane of the fauces, attended with extensive and repeated sloughing of these parts; eruption of the skin, varied in its appearance, form and extent, in different cases, and great debility of the whole of the functions. The affection of the throat, however, was by no means a constant symptom, and the attack was as frequently without it as with it.

"At its commencement, so contagious was the nature of this epidemic to appearance, as to be considered as the epidemic or contagious catarrh combined with quinsy, and in other cases, as measles; and indeed from the very variable mode of its attack, though its nature soon ceased to be in the least doubtful to an experienced practitioner, still it could not fail, from its incipient appearance, to deceive one who looked only to the regular and usual form of scarlatina. In many cases, for three or four days, delirium was the only symptom of the disease, attended with anxiety of the precordia, dyspnœa, palpitation of the heart, cough, bilious vomitings, œdematous swellings of different parts of the body, and, in proportion to the violence of these symptoms, suspension also of sense and motion. In other instances, the malady was ushered in by violent hæmorrhage from the nose and mouth, attended with a quick feeble pulse, and occasionally frequent fits of syncope." P. 111. 112.

It chiefly affected children: we would willingly transcribe this author's very minute account of the symptoms, in the enumeration of which, he has evinced great accuracy of observation; but we have already rendered this article long. The treatment pursued seems to have been judicious, and we were gratified to find that in the cases where cold affusion was freely administered, considerable relief was afforded.

In the autumn, dysentery generally prevails, and is highly contagious. Small-pox was formerly very destructive in this island, but the practice of inoculation has now checked the mortality; and vaccination promises to be successful in further diminishing it. Dr. Gourlay states, that he has not failed in a single instance in producing the disease, and that none of his patients, though afterwards subjected to the contagion of small-pox, have been affected by it.

Measles are frequent among the children in Madeira; the inflammatory flammatory stage is very short, and soon succeeded by a degree of typhoid debility, from which the chief danger arises. The submaxillary glands often swell and prove troublesome. Hooping-cough is epidemic, and the paroxysms are so violent as frequently to terminate in apoplexy. Emetics administered in the commencement of the complaint produce the most benefit.

An appendix contains a short account of the mineral waters in the Portuguese Island of St. Miguel.

Upon the whole we have been gratified by the perusal of this little volume; should a second edition be called for, we hope the author will endeavour to make the department allotted to the natural history of the island more complete. We cannot avoid also remarking, that besides a full page of errata enumerated, various other errors, both of the press and of the editor, have escaped correction.

---

*Remarks on the Nomenclature of the New London Pharmacopœia.* Read before the Liverpool Medical Society. By JOHN BOSTOCK, M. D. &c. Liverpool, 1810. 8vo. pp. 48.

No experiment, perhaps, was ever made more fatal to the high pretensions of corporate bodies in the republic of learning than the late attempts to reform the Pharmacopœias of the three British Colleges of medicine, and justifies the observation with which the author of the pamphlet now under our consideration concludes his preface, that "A royal charter, or an act of parliament, may bestow honours and emoluments, but they are unable to confer knowledge and learning." The failure of these attempts is the more to be regretted, as had they been conducted with liberality, and a real desire for the advancement of science, with which it might have been naturally expected, a priori, these dignified bodies would have regarded the subject, there can be little doubt that medical science, and consequently the community in general, would have drawn the most important benefits from the change. If we may be allowed to judge from the character of the production, we have little hesitation in saying, that a degrading jealousy of each other appears to have influenced all the Colleges; and instead of coalescing to form their Pharmacopœias on a uniform plan, a circumstance the most desirable for the improvement of medical practice throughout the empire, the London College, in particular, appears to have studiously avoided every thing which might have assimilated its Pharmacopœia with those of the Edinburgh and Dublin Colleges, from a truly laudable apprehension, no doubt, of being regarded as copyists.

The

The plan of reforming pharmaceutical language, by employing that of natural history and chemistry, originated with the Edinburgh College. We cannot, certainly, say what would have been the effect, had a communication of the plan been made by that body to the two other colleges, prior to its final adoption; but had it even been rejected by them, the projectors of the reform would have had the pleasing satisfaction of reflecting, that the evils which are now likely to result from the want of uniformity in the three British Pharmacopœias, were in no degree attributable to them. Of the three colleges, however, the London is certainly the most reprehensible, inasmuch as past experience when disregarded must magnify the errors of those who ought to have profited by it: for its production, were we now to go into a comparative criticism of its merits, is more objectionable in the structure of its language, and less scientific in every respect, than either the Edinburgh or the Dublin volumes. But as that is not our present object, we shall now confine ourselves to the examination of the production, the opening of which gave rise to these preliminary reflections.

Doctor Bostock commences by observing, that in his former "Remarks on Pharmaceutical Nomenclature,"* he "attempted to shew, that the advantage to be gained by this assimilation of terms to the language of natural history and chemistry, was more imaginary than real, while the evils to be apprehended from the change, were numerous and considerable." An opinion, in which this second attempt at reform has only tended to confirm him. We must allow his conclusion, although we deny his premises; for although we are clearly of opinion that the assimilation of the language of medical prescription with that of natural history and chemistry is not only attainable to the extent required for practice, but would be highly beneficial; yet, we must admit that the production of the London College has in no degree tended to prove this point. It appears to us that our author has taken too confined a view of the subject, and after minutely examining all the parts in detail, has not reviewed it as a whole by which he might have been led to allow the practicability of the attempt: for it can scarcely be denied, that it would be wrong to condemn the whole of an edifice as bad, because some of the pillars are injudiciously arranged, and some false orders introduced. Even with all its errors we consider the language of the present London Pharmacopœia superior to that of its precursor, the Nomenclature of

* Published by Longman and Co. 1807.

which which was yet undoubtedly more correct than that of the edition which it followed. Indeed, it is probable that the same arguments now employed against any alteration of pharmaceutical language, have been applied to all the changes that have taken place since the publication of the first London Pharmacopœia; and had they been successfully used, we should now be using such terms as *argentum vivum*, *aquila alba*, *vitriolum cœruleum*, *oleum antimonii corrosivum*, *mercurius vitæ*, *sal chalybis*, *terra foliata tartari*, *arcanum duplicatum*, *naphtha vitrioli*, and many others equally barbarous, considering the present advanced stage of science. As an aid to memory, the adoption of the language of modern chemistry, to express the chemical preparations used as remedies, is of itself of much importance; and its want of permanency cannot now be fairly adduced as an argument against it; for the language of pharmacy has at certain distances of time, not very far apart from each other, been varied, and approximating to that assimilation with chemical nomenclature, which has at length been endeavoured, however unsuccessfully, to be accomplished. Nor can we fairly presume to say, that "it will produce little change in the great bulk of medical prescriptions:" as this observation can apply only to old practitioners, who are unacquainted with the new language of chemistry; the change, imperfect as it is, being more congenial to the habits of speech, and the mode of writing of the younger practitioners, and consequently very likely to be readily adopted by them. It is against the ignorance, illiberality, and carelessness of the reformers, not the propriety of the reform, that criticism can successfully aim her shafts, and in this we have every inclination to bear ample testimony to the discrimination and ingenuity with which Dr. Bostock has pointed them.

One of the strongest objections against the introduction of scientific terms, we must admit, is its failure in "producing uniformity in the language of medicine;" and our author has brought forward ample proof of this in the following passage:

"The fact is, that out of between 220 and 230 articles of the Materia Medica, the greatest part of which are the same in both;" (the London and the Edinburgh Pharmacopœias) "there are but 27 to which exactly the same denomination is applied by the two Colleges. Of these 20 retain the names which they had in the former edition of the Pharmacopœia, and one, carbo ligni, is now for the first time introduced into the Materia Medica: so that it is but to six articles that the supposed advantage of uniformity, as a consequence of the introduction of scientific terms, can be attached." (Page 4.)

The Doctor has successfully and properly exposed the futile defence set up by the College, through its agent Dr. Powell, for the adoption of the changes introduced; and were no better reasons to be found, this product of its genius and learning would justly deserve to be neglected; as indeed it would soon of itself naturally fall to the ground, like a soap-bubble blown for the gratification and whim of infantile fancy. But it is fortunate that this defence is unnecessary for the support of the general system, which we are of opinion has sufficient to recommend it, notwithstanding the badness of the execution of some of its parts.

The vague term *abietis resina*, is justly objected to as a word which "means any resinous substance obtained from the spruce fir;" and as being therefore "indeterminate, inaccurate, and only imperfectly scientific:" while *thus*, the former name "was short and distinct, accurately defined and generally understood, sanctioned by use, and conveying no false idea of the nature of the substance." (P. 8.) Acaciæ gummi is also with propriety objected to as a substitute for the well-known term of gum Arabic; first, on account of the uncertainty of the botanical arrangement which has styled the genus in which the plant that is supposed to yield it is now placed: secondly, from the reason there is for supposing that it is the produce of several species: and lastly, with most justice, in our opinion, on account of "the gross, and even dangerous error, of using the generic term alone."

"In the present instance," adds the Doctor, as an illustration of his remark, "the genus Acacia, as constituted by Wildenow, contains no less than 102 species, to each of which the generic term equally applies; so that, were we not previously informed by some other means, what was the article referred to, there is nothing in the name which could enable us to ascertain which of the 102 plants was the one in question." (P. 12.)

A list of sixty-nine articles of the Materia Medica are subjoined in a note, one third of which is clearly liable to the same objection; an error which is the more unpardonable, as some of the members of the Royal College, and we believe one if not two of these were in the Committee for altering the Pharmacopœia, lay claim, and not without valid pretensions, to a high rank in botanical science.

It would be impossible for us to follow the Doctor in each of his remarks, without inserting the whole of the pamphlet into our review; we shall, therefore, notice those only which are remarkable, or to the propriety of which we must object; and point out the omissions, which, notwithstanding the minuteness of the criticism, have escaped our author's observation. Although the names, acetum, ærugo, cerussa, and and such like, are not objected to as improper, inasmuch as they are sanctioned by use; and the components of the substances they are meant to express are either so multifarious, or so insufficiently ascertained, that they could not be named so as to express their composition correctly, or without a long periphrasis, according to modern Nomenclature; yet they are objected to on account of their want of uniformity with the more scientific appellations, and their deviation from the principles on which the College attempted the change. This objection, however, has more plausibility than justice; and does not so much attach to the framers of the Pharmacopœia, as to the insufficiency of modern chemical Nomenclature; of which, however, all that can be said only shews that it is not yet brought to a state of absolute perfection. No objections could have been justly advanced against the language of the Pharmacopœia, had it in all the instances of error erred only in this degree; and we should not condemn the whole of a structure because some of its parts are imperfect. The transposition of the specific names of the salts, as *ammoniæ murias*, instead of *murias ammoniæ*, &c. &c. is justly objected to, as shewing that instead of promoting uniformity, "the framers of the new Nomenclature have seemed rather to seek for opportunities of differing from their contemporaries." (P. 16.) The same force, however, cannot be allowed to the objection which our author states against the retention of the old name for gum ammoniac; for although Wildenow does not doubt that this substance is obtained from the heracleum *gummiferum*, a plant which he raised from seeds found in the gum ammoniac of the shops, yet he has not succeeded in obtaining any gum-resin from the plants he reared; and the little reliance which can be put on the unscientific description, and engraving, of the plant named *ferhaale*, from which the Barbary ammoniacum is obtained, given by Mr. Jackson in his History of Morocco, would not authorize the College in adopting the opinion of even so respectable a naturalist as Wildenow, till the identity of the plants be more accurately established. The very puerile alterations in the orthography of the words columba and gambogia, which are now spelt calumba and cambogia, are justly ridiculed; but our author is wrong in objecting to the part of the capsicum plant which is used, being styled bacca, and stating, that "it is not the *berry*, but the *capsule* that is employed in medicine." If this passage means to imply that the fruit of the capsicum is improperly styled a *berry*, the College may urge the authority of Gærtner, the highest on this subject, who styles it, in his description, "Bacca cava, multiformis:" but even if it

 means, means, as we suppose to be the case, that the outside of the berry or the covering part only of the fruit being used, the word berry, which designates the whole of the fruit, should not be employed, the term *capsule* would not be correct in this case, as it properly signifies a particular kind of fruit. The truth is, the part medically employed is the *pericarp*, and hence it should have stood in the Pharmacopœia *capsici annui pericarpium*. We cannot defend the College for the adoption of the term cetaceum, instead of spermaceti; but it may, perhaps, be allowed the new name does not convey an erroneous idea of the substance, which was the case with the old term, although sanctioned by long usage.

Doctor Bostock points out as deserving of particular notice, the frequent alteration which the names of the three kinds of cinchona have experienced; and properly observes that the use of the specific names of the plants, instead of the old titles of the barks, "are a mere exercise of the memory, while the old ones could scarcely be either mistaken or forgotten." The objection to this mode of naming the bark is, in our opinion, not pushed sufficiently far; for there seems to be a misunderstanding between Zea, on whose authority the change is made, and the College, at least if we can refer to the translation of Dr. Powell, its accredited translator. In the translation, the yellow bark of the shops is regarded as the heart-leaved cinchona bark, cinchonæ cordifolia cortex, whereas Zea states the cordifolia to be the species which yields the quina amarilla of the Spaniards, which is the common pale or officinal bark; while the cancifolia, which Doctor Powell regards as yielding the pale bark, yields, according to Zea, the quina naranjada of the Spaniards, the calisaga of commerce, which is the yellow or orange bark of the shops. The improper application of general terms to imply particular substances, as cornua, to express the horns of the stag; ovum, the hen's egg; sevum, mutton suet; and testæ, oyster shells; is very properly condemned. The objection to saccharum, which is used to express the sugar prepared from the arundo saccharifera, because it "is the name of a class of vegetable products," is completely hypercritical; and the same may be said of our author's objection to the term, camphor. An unintentional mistake occurs in stating the impropriety of the appellation lytta. After stating the change made by the Edinburgh College in discarding the title cantharis, and adopting that of meloe vesicatorius; "this," says the Doctor, "however, from the change of system, or from new discoveries in natural history, is destined to give way to the still more novel appellation of lytta vesicatoria; of which, although there are twenty-nine species, species, and the specific name here has been improperly added, for lytta vesicatoria is but one species; and had it so stood in the Pharmacopœia instead of the simple generic term, lytta, there could have been no reasonable objection made to the change, nor would the name have been as it now is, "imperfect and indeterminate." We cannot allow that the same objections which are correctly stated to the chemical synonymes of verdigris, and white lead, apply to litharge. In our opinion the name plumbi oxydum semivitreum, adopted by the College, is perfectly correct; for the small portion of carbonic acid it contains cannot be regarded as sufficiently essential to constitute it a subcarbonate. The number of instances, however, in which the name adopted by the college is completely at variance with the synonyme in the opposite column is very remarkable; our author has properly pointed out all of these; and in particular considers "the change of the old word borax to boras-sodæ, is highly censurable, as not only being an incorrect expression, which seems to have been designedly adopted, but as properly belonging to a different substance." P. 21. In this and similar instances it is almost impossible to conceive the motives of the College; for the synonyme is generally a severe criticism on the name of the substance: thus, in the present case, in the first column of the list of Materia Medica is placed "Sodæ Boras," while opposite, in the second column, we find the proper name of the salt, "subboras sodæ." We think there is some reason for the retention of the term spiritus rectificatus, to which Dr. Bostock objects, the term alcohol being employed to designate a purer spirit, of a much less specific gravity; and as there is still a weaker spirit than the common rectified spirit in use, the adjunct *dilatus* would not have been sufficient to have pointed out the distinction between these; while by the terms used by the College, the three degrees of strength are fully specified. As gum arabic has been rejected, the term tragacantha can scarcely be admitted as a proper appellation for the gum of the astragalus vera; and therefore, our author's objection to it is perfectly valid.

The remarks on the second part of the Pharmacopœia are equally just as those we have already noticed; and many of them expose a degree of neglect, for it cannot be termed ignorance, of the College, which is extremely culpable. But as we before observed, were we to follow the Doctor step by step, we should be obliged to embody the whole of the pamphlet in our critique; we shall, therefore, only enumerate those titles, which are considered as objectionable. *Ammoniæ carbonas, liquor ammoniæ carbonatis, soda tartarizata,*

*sulphuretum*

*sulphuretum antimonii præcipitatum*, *antimonium tartarizatum*, *pulvis antimonialis*, *argenti nitras?* This preparation Dr. Bostock regards as a subnitrate, and, therefore, affirms that it should have been named *subnitras argenti:* but his opinion in this instance may be questioned, for the silver as held in solution in the nitric acid, in the preparation of the London College, is evidently an oxynitrate; it is also in the same state in the salt obtained by evaporating the solution; and, although, by the subsequent melting a part of the acid be expelled, yet, it is very probable that the product is not reduced to the state of a subnitrate. *Liquor arsenicalis*, *cuprum ammoniatum*, *ferrum ammoniatum*, *ferri carbonas*, *ferrum tartarizatum*, *liquor ferri alkalini*, *tinctura ferri muriati*, *vinum ferri*, *hydrargyri oxymurias*, *hydrargyri submurias*, *hydrargyrus cum creta*, *hydrargyri oxydum cinereum*, *hydrargyrus præcipitatus albus*, *liquor aluminis compositus*, *decoctum aloes compositum*, *infusum catechu*, *infusum sennæ*, *tinctura camphoræ composita*, *extractum colocynthidis compositum*, *mistura ferri composita*. Notwithstanding the extent of the above list, added to the objectionable names pointed out by Doctor Bostock in the first part, there are still others which he has overlooked, and which we shall therefore now mention. The part of the bulbous plants employed is invariably denominated *radix* instead of *bulbus;* the fruit of momordica elaterium is improperly termed *pomum*, instead of *pepo;* to *acidum aceticum*, the word *dilutum* should have been added, as it is meant to imply distilled vinegar; *magnesiæ carbonas* should be *subcarbonas magnesiæ; decoctum cydoniæ* should have been *decoctum cydoniæ seminum;* and *decoctum lichenis* should have added to it the specific appellation of the plant *islandicus*, the lichen rocella being now admitted as an article of the Materia Medica by the Dublin College. Doctor Bostock objects to the term *oxydum antimonii*, not so much on account of the name, but on account of its being improperly given as a synonyme of *antimonium calcinatum*, *antimonium vitrificatum*, and *crocus antimonii:* from our experiments we have no hesitation in saying that, if the preparation which is perfectly useless be allowed to remain, it should be named *submurias antimonii.*

Upon the whole, although we do not agree with our ingenuous author in deprecating the principle of the change of Nomenclature altogether, yet, we cannot withhold from him the tribute of applause for his acumen, and the propriety of his remarks on the execution of the production of the London College. We regret most deeply the circumstances which have produced these errors; particularly at the present sent moment; for, when the cry against irregulars is sounded so loudly over the island, we should at least expect to find, if not infallibility, a degree of correctness and knowledge commensurate with the high pretension of the regularly initiated, the *perhonorifici mystagogi* of the Warwick Lane Temple.

---

### *Edinburgh Med. and Surg. Jour.*

(Concluded from Page 360.)

### Art. VI. *Description of a very singular and complicated Case of Malconformation of the Genital Organs, Rectum, &c.* By T. Conquest, Surgeon.

"The whole body of the child was emaciated, and great deficiency of vigour was perceptible; the lower extremities were considerably distorted, the feet being turned completely inwards. On the second and third lumbar vertebiæ, there was a *spina-bifida* of the size of a common orange.

"The *umbilicus* was lower than usual, and on the right side; it did not appear to perforate the linea alba in its natural place, but came out nearer the rectus muscle; there was a considerable elevation, both on the upper and lower part of its perforation.

"The *anus*, in its natural situation, was imperforate, nor was any appearance of the termination of the rectum perceptible, when exertions were made, by urging or voiding the urine. About midway between the umbilicus and symphisis pubis, the rectum protruded about three inches from the abdomen, and was covered by the common integuments, which were regularly continued on from the abdomen, and reflected over its whole length. The peristaltic motion was constantly going on, and the meconium were (was) voided through it. It was much thicker than the rectum of an adult, supposing it to have the addition of a cuticular covering. It appeared to be surrounded with a strong muscle, which, when the internal or villous coat suffered the least attrition, contracted with an unusual degree of force, amounting to a temporary obliteration of the canal. The sphincter was perfect at its termination, where it was for a small distance inverted. The inversion of its lower part rendered the internal coat so distinct, as to admit of being minutely examined. The villous coat was extremely red, vascular, and tinged with blood of a florid hue. It was so extremely sensible, that when slightly irritated with a probe, a determination of blood took place to the part, and the peristaltic action increased almost to an erection; it was studded with innumerable and very large glands, which secreted a large quantity of mucus.

"*The genital organs* presented an appearance equally singular with that of the parts already described. Their structure was so complicated, it was almost impossible to form any correct opinion of the the sex; neither predominated, and there appeared an admixture of both. That which would at first sight have been pronounced to be a scrotum, was considerably larger than one of the common size. It extended above the pubis, and was smooth on its surface, without the least appearance of raphe, nor was any perinœum to be distinguished. Nothing which bore the least resemblance to testis could be discovered, the scrotum or tumour was filled with cellular substance, of a spongy texture. On the most minute examination, it evidently was not an adhesion of the opposite sides of the labiæ. On the upper part of this tumour was a small prominence, perforated with a fissure, so small as to render the introduction of a probe impracticable: this was the orifice of the urethra, and bore a near resemblance to the meatus urinarius; it had no appearance of prepuce, and was not in any respect similar to the penis. The urethra could be traced through the soft cellular substance, with which the tumour was filled as far as the symphisis pubis; it was unusually hard and felt like a curved probe. There was a constant involuntary discharge of urine, which exuded from the fissure *guttatim*, except on any violent exertion of the child, when it flowed in an excessively fine stream or jet. In cases which have been described in some respects similar to this, a separation of the bones of the pubis has been mentioned; but in this case there was no appearance."

---

### Art. VII. *Sketch of a Plan for improving the Medical Department of the Navy.*

Earnest as we have always been in recommending an assiduous attention to the collecting of facts, which we have considered as materials hereafter to be used in the construction of a true system of medicine, and as the foundation of a practice that may, possibly, at some future period, overcome diseases now intractable; we cannot but give our approbation to the comprehensive views of this anonymous writer. At the same time we must observe, that they require a too constant attention ever to be generally executed, except under the direct command of the government.

After some preliminary observations following a very appropriate motto from Horace,

"Tu, dum tua navis in alto est,

Hoc age." ————————

"Hoc opus, hoc studium parvi properemus et ampli,

Si patriæ volumus, si nobis vivere cari."

The writer proceeds to state the outline of his plan.

"I would have him (the naval surgeon) consider the body of men, of which he is the medical superintendant, as the subject of an experiment placed within the influence of all external bodies. I would have him mark the changes which these reciprocally undergo, endeavouring, with with unceasing attention, to ascertain the causes of the different changes that arise on this subject of experiment, and to explain the mode of action of which these are the result. When the effects are *disease*, he is to note its whole phenomena, still remarking, in a particular manner, its relation with external bodies, and the effects of these on the subject in this altered condition. In other words, I would have the surgeon to pay particular attention to the whole ship's company, as well in health as in disease, observing and marking their mode of living, as to food, drink, clothing, exercise, &c.; their different conditions and habits, and the effects of these on health and disease; particularly noting the state of the weather, and endeavouring to ascertain its influence. When disease occurs, he is to pay great attention to every case, so as not merely to benefit his patient as much as possible by the best practice, but to instruct himself from every additional sample of disease.

"All this is a matter of the easiest attainment, and may be very well accomplished in the following manner: In a journal, kept for the purpose, the surgeon is to commence by inserting a list of the ship's company, as divided into the different classes of seamen and marines, subdivided into forecastle men, top men, afterguard, &c.; in short, into as many divisions as different occupations may be supposed to give rise to difference in health. After every name (as obtained from particular inspection, observation, and enquiry) the man's appearance or temperament, habits, character, nature of employment, years at sea, former condition of life, previous state of health, &c. are to be marked; particular attention being paid to every thing which may be supposed to influence health.

"In another part of the journal he is daily to note the state of the weather, as ascertained from general observation, and from instruments. In addition to the account of the state of the atmosphere, the particular state of things, as shewn locally between decks, &c. as with regard to moisture, air, &c. is to be mentioned. Then are to be marked the nature of the duty carried on, and the diet and drink of the ship's company for the day. When any uncommon or very particular duty is carried on by a small number, such as wooding, watering, &c. the names of the men employed are to be marked in the daily reports, and the particular nature of the duty to which they have been subjected, detailed. Then the sick-list is to be reviewed, and the number and names of the men and diseases admitted and discharged are to be marked. At convenient times, the general character of the prevailing diseases is to be inserted, referring for a detail of the particular cases to another journal. *This other journal* is to contain a detail of every case of disease which is entered on the list: not a cursory enumeration of the most prominent symptoms, as caught from the first glance at the patient, but a regular and leisurely account of all the *complaints* of the patient, *the present state* of the *usual criteria* of disease, the medicines *taken*, and their *evident* effects; in short, a true and full history of the disease, from which may be learned the actual state of the patient's system, and a portable opinion formed of the effects of of the remedies employed. This account is to be taken at stated times (once or twice a day) and fully given at each time.

"The plan now proposed, carried on privately, would doubtless contribute greatly to individual as well as to general improvement; but it would be far from producing all the great effects to be expected from it, were the perfection of it left to the volition of individuals, or to observations formed from partial reports. To accomplish the end in view, it would be necessary that surgeons should be obliged, by their instructions, to transmit the two journals above mentioned, at stated times, to the board, or to some persons appointed for the purpose. The duty of these would be to collate the immense body of evidence, to condense the general facts, and, in short, to reduce to a manageable volume, the whole of the facts and observations contained in the journals, either drawing from these the general results, or leaving this to the discretion of the reader. This might be published annually, or at any convenient times, and would be drawn up in whatever form might appear most advantageous for purposes of general improvement. The gentlemen, whom the nature of their situation points out as best qualified for this task of arrangement, are the physicians of the different fleets. They being on the spot where the observations were made, and having probably visited every ship, have on many accounts advantages which none others can possess. Each physician would collect the reports of his own station; and thus we would have annual reports of the Channel, North Sea, Mediterranean, West India, and East India stations. Reducing the magnitude of the undertaking, we would expect the reports to be more correct; at the same time we would have a better opportunity of comparing the effects of the different climates.

"The advantages resulting from a faithful and persevering prosecution of this simple plan, it is evident, would be great. Let us suppose that a suite of remarks and observations on the above plan was carried on for a few years, say ten only, by seven hundred surgeons, in vessels of every class, in all variety of circumstances, in every climate of the globe; that these remarks and observations were compared and collated by some person capable of the undertaking, in the manner it has been proposed. Need I waste time in stating, what must appear at first view to every one, the valuable acquisition such a work would be to medicine in general, and to nautical medicine in particular? But leaving such bounded views, when we consider the effects of this plan, faithfully pursued for centuries, amended by the progressive increase of improvement, and enriched by the discoveries of so many years, we are surprised at the immense views of improvement that open on us, and cannot help regretting the inactivity of man, and blaming the indolence and carelessness of our predecessors, who have left a work of such mighty import for the *speculation only* of the present day."

Art. VIII. *Account of two Cases of Gonorrhœa, attended with unusually severe Symptoms, relieved by the employment of Calomel and Opium in combination.* By Mr. Langstaff, Surgeon.

In the first of these cases, the inflammatory symptoms, the distention of the penis, and the suppression of urine, were most alarming. Many methods were had recourse to, with temporary relief only: at length a combination of opium and calomel, in equal parts, was given in the quantity of two grains of each, night and morning, with the most evident benefit. Opium by itself did not relieve the disease, but when combined with calomel, its effect was quick and decided. The other case of suppression of urine, relieved by opium and calomel, is given from Dr. James Curry. The effect said to be produced by this combination of two active substances, is of sufficient importance to excite the attention of the faculty.

---

Art. IX. *Remarks on Sore Nipples.* Anon.

This writer proposes a prophylactic for this distressing complaint. The shield and teat so commonly used in and about London, is the means he recommends to secure the mother. The shield may be had at the instrument makers, either in silver or ivory. The artificial nipple is the teat of a fresh slain heifer. This is prepared by carefully scooping out the inside, so as to leave the teat about the thickness of Morocco leather. The operator should be particularly careful that he does not make the smallest puncture in the skin of the sides of the teat; for if this happens it will not be air tight, and of course useless. The teat thus prepared, after being well washed in water, must be kept in spirit, till an hour or two before using, when it must again be laid in cold water to take away all taste of alcohol. Being wiped perfectly dry, it is to be sewn closely and firmly at the edge of the row of holes in the shield, and need not be removed from the shield until it becomes bad from use. The whole apparatus must be carefully washed after suckling, and kept constantly in cold water; or if the weather be hot, in spirit and water, with the precaution, in the latter case, of rinsing well before using.

ART. X. *An additional Account of the lythontriptic Power of the muriatic Acid, with an Analysis of the calculous Matter discharged in one Case, during its Use; also an Account of the lythontriptic Power of the nitrous Acid, with an Analysis of the calculous Matter discharged in a Case during its Employment.* By PETER COPLAND, Little-Bytham, near Stamford.

THE attention of Mr. Copland to the lithontriptic properties of the muriatic acid, was first excited by cases of its successful employment, related in the fifth and sixth volumes of the Memoirs of the Medical Society of London.

The cases here detailed afford sufficient evidence to induce a farther trial of the muriatic and nitrous acids in calculus. In the first, thirty drops of the muriatic acid were taken in water three times a day; the dose was gradually increased to fifty drops, and continued till two ounces were taken, when the complaint was removed. The patient was directed to collect daily, in one vessel, all the urine which could be obtained in twenty-four hours. The clear urine was then poured off, and the sediment collected upon a paper filtre. The sediment thus collected amounted to 104 grains, of a buff coloured impalpable powder.

One hundred grains of this powder subjected to chemical analysis, was found to contain

| | Grains. |
|---|---|
| Uric acid . . . . . . | 72 |
| Ammonia . . . . . . . | 18 |
| Carbonate of lime . . . | 3 |
| Phosphate of lime . . . | 5 |
| Loss . . . . . . . . . | 2 |
| | 100 |

In the other case, (that of Sir Simon Kelly, of Colsterworth, Lincolnshire) forty drops of the diluted nitrous acid were taken in water every two hours, till a sediment appeared in the urine; and afterwards continued four times a day, while necessary. Twenty-seven ounces of the diluted acid were used. A sediment soon appeared, and by persevering with the medicine from November 12, 1805, to April 20, 1806, 600 grains of a light thick coloured powder were collected; in which, toward the conclusion, a few fragments of calculus calculus were found, partially decomposed. One hundred grains of this sediment, subjected to analysis, gave

| | Grains. |
|---|---|
| Uric acid . . . . . . . | 80 |
| Ammonia . . . . . . . | 11 |
| Carbonate of lime . . . . | 2 |
| Phosphate of lime . . | 1 |
| Loss . . . . . . . . . | 6 |
| | 100 |

During the treatment of these cases opium was occasionally given to mitigate pain; costiveness was prevented by mild laxatives; and when the stomach was oppressed by the frequent doses of the acid, it was relieved by taking any convenient spirit diluted with water. It appears, from this gentleman's observations, that the *nitrous acid* possesses greater lithontriptic powers than the *muriatic*: and that in many cases of *lithiasis*, it always procured a discharge of sediment with the urine; and a due perseverance in its use was followed by a removal of the symptoms.

---

ART. XI. *An Account of the Larvæ of two Species of Insects discharged from the human Body*. By T. BATEMAN, M.D. F.L.S.

As a record of facts, on a subject of considerable interest in the sciences of medicine and natural history, Dr. Bateman's paper may be considered as valuable. Whoever adds one unequivocal truth to a department in science, abounding with, at least, doubtful histories, may be considered as the benefactor of mankind.

Without having recourse to the animalcular hypothesis to account for fever, &c. it cannot be denied but painful, and even fatal cases, have arisen from the existence of the *larvæ* of insects in different parts of the human body.

In the first case related, the *larva* of the common black beetle (*tenebrio molitor. Lin.*) was discharged from the bowels: in the second, the *larva* of the common house-fly (*musca domestica minor. De Geer*), was thrown up from the stomach by vomiting.

Dr. Bateman accompanies his details with a general view of the subject, as found in the records of natural history and medicine, and with observations on mistakes which have often misled the physician.

ART.

Art. XII. *Case of Fungus Hæmatodes.* By John Henry Wishart, Surgeon.

This disease occurred in a child three years old. When Mr. Wishart first saw the patient there was a tumour as large as the fist, situated on the right side of the head, surrounding the ear, and extending from the zygomatic process downward on the anterior side of the neck. This tumour was soft and inelastic; the integuments covering the part anterior to the external ear, were of a whiter colour than natural, but immediately behind the under part of the ear they had ulcerated, and a small fungus was observed to protrude, discharging a thin fœtid sanies, frequently mixed with blood, and occasionally a slight hemorrhage occurred. A brownish substance protruded from the meatus externus, resembling in colour the surface of the ulcerated part.

From the 10th of August, the time when Mr. Wishart first saw the case, the swelling increased rapidly; a livid spot ulcerated three weeks after its appearance, a fungus grew up from this spot, which frequently sloughed off, but was quickly regenerated, and discharged a large quantity of fœtid bloody matter, and frequently hemorrhage to the extent of an ounce or two occurred. The symptoms became daily more severe, accompanied with pain, which increased with them until the 22d of September, when the child died, exactly fifteen weeks and four days after the appearance of the disease.

The tumour was examined after death. It very much resembled the engraving of Mr. Astley Cooper's case in Mr. Wardrop's work on Fungus Hæmatodes; was as large as the child's head, and weighed forty ounces. It was a fungous mass, soft and friable, had a most offensive smell; and a slice of it compared with a slice of the brain, was in appearance so similar, as hardly to be distinguished from it. An aperture was observed in the squamous portion of the temporal bone, through which a small part of the tumour had passed inward, and formed a depression on the middle lobe of the brain, about half an inch in depth. This portion of the tumour was the size of a small egg, of a round shape, connected with the external tumour by a narrow neck, and did not adhere to the brain. The dura mater was destroyed at the part where the depression was formed; in other respects the brain was sound.

It is remarkable that the patient's general health was but slightly deranged; for during the progress of the disease, till within within a few days of his death, he slept well, had no thirst, heard distinctly, and even more acutely, it was believed, on the diseased side; vision was unaffected till the four last days of his life; he was never observed to be comatose, and could move the upper and lower extremities frequently.

---

Art. XIII. *Case of præternatural Anus, found in a Portion of Ilium protruded at the Umbilicus.* By J. Peake, Surgeon.

A Child was born with a portion of ilium protruding at the umbilicus, in which was an external opening. Through this passed some meconium, as it did also by the natural anus. The child lived two days.

---

Art. XIV. *Appearance upon Dissection of two Dogs, which were killed while labouring under Rabies.* By the Same.

No fact relative to the nature or cause of *rabies canina* should pass unnoticed. Upon this principle it is, that we insert the appearances discovered by Mr. Peake, on the dissection of those dogs. It is to be wished that we had undertaken his examination unbiassed by the opinion that the disease is seated in the mucous membrane of the œsophagus and stomach.

Upon opening the stomach of the first dog, it was found to be nearly empty; containing only a small quantity of short oat straw, some grit, and a portion of gastric juice. The mucous membrane was inflamed at different parts, both in the stomach and œsophagus. The intestinal canal was nearly empty, and of its natural appearance.

The stomach of the second dog was found much distended with pieces of straw and hair, and some grit or earth. Its mucous membrane appeared slightly red and inflamed generally; but at the extremity, near the pylorus, was a greenish spot about the size of a shilling, with a narrow line of the same colour, extending upward; the result, as it appeared to Mr. Peake, of preceding inflammation. The œsophagus was of its natural colour.

---

*Cursory Remarks on Corpulence.* By a Member of the Royal College of Surgeons. 8vo. London. 1810. pp. 44.

That *Obesity* is ever an actual disease, if we are not prepared to deny, we cannot fully admit. When it exists to a certain degree, though it may hardly be called a morbid state state of the system, yet it may oppress some organs so much, and blunt their functions so effectually as to render life burthensome, if it be not destructive to vitality. Whether in the strictness of language a natural action of the system, and which is peculiarly energetic in the most healthy state, can be considered as a disease, might be disputed, even though nosologists have given it a place in their systems under the generic term *Polysarcia*. Be this as it may, physicians have not been inattentive to its progress, and have stated with great precision, some instances of the excessive increase of adipose substance. In the Transactions of the London College of Physicians, the case of Thomas Wood, a miller, of Billericay, in the county of Essex, affords a remarkable instance of the inconvenience of corpulency, of a train of morbid effects apparently connected with it, and of as remarkable a relief from alarming symptoms, by persevering in a most abstemious regimen. In his 40th year, Wood began to grow very fat. In his 44th year he became disturbed in his sleep, complained of heart-burn, of frequent sickness, pains in his bowels, head-ach, and vertigo. He was sometimes costive, and at other times in the opposite extreme; had almost a constant thirst, great lowness of spirits, violent rheumatism, and frequent attacks of gout. He had likewise two epileptic fits. But the symptom which appeared to him to be the most formidable, was a sense of suffocation, which often came upon him, particularly after meals. Whether these are the symptoms that arise out of a sudden accumulation of fat, we cannot determine; but certain it is Wood was relieved from them all by a course of rigid abstinence. He relinquished animal food, eat nothing but pudding made with flour and water, drank no kind of fluid. Under this spare diet his complaints vanished, his size was reduced, and he became chearful and alert.

The third volume of the Medical Observations gives an interesting case of a man who died from an enormous increase of internal *adeps* without its existence being manifested by any great degree of external corpulence. The symptoms were singularly anomalous, and the inspection of the body ascertained that a large mass of fat filled the upper part of the breast; that in the abdomen there was an amazing collection of adipose substance, not only in the omentum, but in the mesentery and mesocolon, where not a vestige appeared of blood-vessels, glands, &c. which all were buried in a prodigious heap of fat. This fat had no where formed a preternatural tumour, or changed its consistence or colour, but was accumulated in an unusual quantity wherever it is naturally rally lodged more or less in subjects well constituted ; it only appeared a little less solid, and with a tinge somewhat yellower than in its natural condition. In the lower cavity this mass compressed the stomach, and hindered the action of the lungs; and in the posterior mediastinum it surrounded the œsophagus from the pharynx to its passage out of the breast, variously affecting deglutition. The bed of adeps under the upper part of the sternum was so considerable, that this alone would have been sufficient to obstruct many of the functions of that cavity. The fifth volume of the work last cited, contains some interesting particulars respecting diseases, that seem to arise out of an extraordinary obesity. Among these its tendency to produce angina pectoris deserves particular notice. In 1727, Dr. Thomas Short published a pamphlet on Corpulency; and about 1760, Dr. Malcolm Flemyng, a physician of some notoriety, and an author of repute, both as a theorist and a practical writer, published a Discourse on the Nature, Cause, and Cure of Corpulency, and against which he considers soap to be a sovereign remedy.

The little pamphlet before us, which is written with a degree of neatness, though a mere compilation, details the opinion of various authors on the treatment of this state of the system. Cœlius Aurelianus recommends abstinence and exercise, reading aloud, friction on the skin with sand, and sweating in the *sudatorium.* Zacutus Lusitianus employed leeches and scarifications ; Fernelius diuretics. Borelli advises chewing tobacco, and Etmuller taking vinegar of squills. Dr. Darwin believes salt eaten in considerable quantity, or salted meat, to be more efficacious than soap. Dr. Beddoes asserts the cure of obesity is effected by the abstraction of oxygen from the constitution. But the surest way is that which was pursued by Wood : abstinence and exercise reduced the Essex miller to a moderate size. It is a curious coincidence, that the fattest man before the time of Lambert, in this country of fat men, should have lived in Essex. Bright, of Walden, weighed thirty-two stone ; but Lambert is said to have exceeded this enormous weight by twenty stone.

What diet is most likely to produce corpulence, is a question of some importance. M. Lorry (Memoires de l'Academie de Medicine, where he gives an account of diseases supposed to be produced by corpulence) considers the abundant use of succulent vegetable aliment to certainly produce this state of the system ; and the following case, with which we shall conclude these observations, goes strongly to establish this point.

"A man of about forty years of age hired himself as a labourer in one of the most considerable ale-breweries in the city: at this time he was a personable man; stout, active, and not fatter than a moderate sized man in high health should be. His chief occupation was to superintend the working of the new beer, and occasionally to sit up at night to watch the wort, an employment not requiring either activity or labour; of course at these times he had an opportunity of tasting the liquor, of which it appears, he always availed himself; besides this, he had constant access to the new beer. Thus leading a quiet inactive life, he began to increase in bulk, and continued to enlarge, until, in a very short time, he became of such an unwieldly size, as to be unable to move about, and was too big to pass up the brewhouse staircase: if by any accident he fell down, he was unable to get up again without help. The integuments of his face hung down to the shoulders and breast: the fat was not confined to any particular part, but diffused over the whole of his body, arms, legs, &c. making his appearance such as to attract the attention of all who saw him. He left this service to go into the country, being a burthen to himself, and totally useless to his employers. About two years afterwards he called upon his old masters in a very different shape to that above described, being reduced in size nearly half, and weighing little more than ten stone. The account that he gave of himself was, that as soon as he had quitted the brewhouse he went into Bedfordshire, where having soon spent the money he had earned, and being unable to work, he was brought into such a state of poverty, as to be scarcely able to obtain the sustenance of life, often being a whole day without food; he drank very little, and that was generally water. By this mode of living he began to diminish in size, so as to be able to walk about with tolerable ease. He then engaged himself to a farmer, with whom he staid a considerable time, and in the latter part of his service, was able to go through very hard labour, sometimes being in the field ploughing and following various agricultural concerns, for a whole day, with no other food than a small pittance of bread and cheese. This was the history he gave of the means by which this extraordinary change was brought about. He added his health had never been so good as it then was."

