# Corrigenda

**Published:** 1811-06

**Authors:** 


					P. 436, 1. 20, dele the word of.
437, 1- 33, for ferhaale, read Feshooh.
438, 1. 22, for cordifolia, read cordifolice.
438, 1. 28, for cancifolia, read lancifolia.
438, 1. 31, for calisaga, read calisaija.
439, 1.31, for dilatus, read dilutus.
451, 1. 36, for Wuldeti, read Maiden.

				

